# ‘I won’t make it without this program’: the impact of safer opioid supply program closures in Ontario

**DOI:** 10.1186/s12954-025-01298-6

**Published:** 2025-09-24

**Authors:** Farihah Ali, Abigale Sprakes, Jordan Mende-Gibson, Cayley Russell, Andrew Shaw, Matthew Bonn, Andrzej Celinski, Nat Kaminski, Mohammad Karamouzian, Carol Strike, Jürgen Rehm

**Affiliations:** 1 Institute for Mental Health Policy Research, Centre for Addiction and Mental Health (CAMH), Toronto, M5S 2S1 Canada; 2 Ontario Node, Canadian Research Initiative in Substance Matters (CRISM), Centre for Addiction and Mental Health (CAMH), Toronto, M5S 2S1 Canada; 3 Dalla Lana School of Public Health, University of Toronto, 480 University Ave, Suite 300, Toronto, ON M5G 1V2 Canada; 4 Campbell Family Mental Health Research Institute, Centre for Addiction and Mental Health, 250 College St., Toronto, ON M5T 1R8 Canada; 5 ChangeMark Research & Evaluation, Vancouver, BC Canada; 6 Workers for Ethical Substance Use Policy (WESUP), Vancouver, BC Canada; 7 Moyo Health & Community Services, 7700 Hurontario St #601, Brampton, ON L6Y 5B5 Canada; 8 Peel Drug Users Network (PDUN), Brampton, ON Canada; 9 Faculty of Health and Behavioural Sciences, School of Social Work, Lakehead University, 955 Oliver Road, Thunder Bay, ON P7B 5E1 Canada; 10 Centre on Drug Policy Evaluation, MAP Centre for Urban Health Solutions, St. Michael’s Hospital, Toronto, ON Canada; 11 RECLAIM Collective, Toronto, ON Canada; 12 Department of Psychiatry, Faculty of Medicine, University of Toronto, 250 College Street, 8th floor, Toronto, ON M5T 1R8 Canada; 13 Faculty of Medicine, Institute of Medical Science, University of Toronto, Medical Sciences Building, 1 King’s College Circle, Room 2374, Toronto, ON M5S 1A8 Canada; 14 Center for Interdisciplinary Addiction Research (ZIS), Department of Psychiatry and Psychotherapy, University Medical Center Hamburg- Eppendorf (UKE), Martinistraße 52, 20246 Hamburg, Germany; 15 Program on Substance Abuse and WHO European Region Collaboration Centre, Public Health Agency of Catalonia, Aragó Street 330, 08009 Barcelona, Catalonia Spain; 16 Faculty of Applied Health and Community Studies, School of Applied Health, Clinical Research, Sheridan College, 7899 McLaughlin Rd, Brampton, ON L6Y 0P8 Canada

**Keywords:** Safer opioid supply programs, Harm reduction, Ontario, Overdose risk, Opioid crisis, Policy changes

## Abstract

**Background:**

Canada is in the midst of a worsening overdose crisis, driven largely by the unregulated drug supply. In response, safer opioid supply (SOS) programs were implemented to provide pharmaceutical-grade opioids alongside critical services. However, in August 2024, Ontario’s provincial government introduced restrictions on harm reduction initiatives, coinciding with the expiration of federal funding, forcing many programs to close. In their place, the government announced the implementation of Homelessness and Addiction Recovery Treatment Hubs, which exclude harm reduction programs, including SOS programs. This study explores the experiences of SOS program clients and the anticipated impacts of these program closures on their lives.

**Methods:**

A qualitative study design was used, involving semi-structured interviews with people who use drugs who were enrolled in six SOS programs across Ontario. Participants were recruited through convenience and snowball sampling. Eligible participants were current SOS clients (≥ 6 months), aged 18 or older, and English-speaking. Interviews were conducted virtually and explored participants’ experiences with SOS programs, anticipated impacts of program closures, and strategies to mitigate risks. Data were thematically analyzed using NVivo.

**Results:**

Participants reported that SOS programs reduced their reliance on the unregulated drug supply, decreased their overdose risk, and connected them to wraparound services. The impending closures triggered widespread fear, uncertainty, and anxiety, particularly about returning to the unregulated supply. Participants also expressed concerns over the loss of access to critical health and social services and the potential decline in their quality of life. Many expressed frustration over the lack of meaningful alternatives, difficulties in securing new prescribers, and distress over forced medication tapers.

**Conclusion:**

This study highlights participants’ concerns that SOS program closures may force them back into an increasingly dangerous unregulated market, ultimately putting their lives at risk, along with reversing the many benefits SOS programs provided, such as connections to essential health and social services. By replacing harm reduction programs with treatment services, the government is not reducing the demand for opioid use; instead, it forces a return to the unregulated drug market, ultimately putting individuals at risk of overdose.

## Background

The overdose crisis in Ontario has escalated significantly up to 2023, claiming more than 16,000 lives since 2016 [1]. The increasing prevalence of fentanyl, nitazenes, and novel benzodiazepines, as well as potent veterinary compounds, such as xylazine and medetomidine, in the unregulated drug supply has placed people who use drugs at heightened risk of fatal and non-fatal overdoses [2–4]. In response, harm reduction initiatives such as prescribed safer supply programs (SSPs), also known as safer opioid supply (SOS) or the prescribed alternatives program, have been funded nationally as part of the Canadian Drugs and Substances Strategy (CDSS) [5] to address the drug toxicity crisis [6]. These programs are designed to reduce reliance on the toxic unregulated drug supply by providing pharmaceutical-grade substances, such as opioids, stimulants, and benzodiazepines, as safer alternatives [6].

In Canada, federally funded SOS programs were first introduced in British Columbia and Ontario, before expanding to other provinces through the federal government’s Substance Use and Addiction Program (SUAP) [7]. These programs offer medical-grade opioids under prescribing models and are typically integrated within community health centres that provide wraparound health and social services to support people who use drugs [7–12]. Some of these organizations include Supervised Consumption Services (SCS), known in Ontario as Consumption and Treatment Services (CTS) [13]. SOS programs complement other interventions, such as opioid agonist treatment (OAT), needle exchange programs, and housing-first models [7], offering a critical access point for people who use drugs to engage with healthcare, harm reduction services, and social support services [7, 8]. SOS programs have played a vital role in mitigating the devastating impact of the overdose crisis, particularly as the toxicity of the unregulated drug supply continues to rise. These programs contribute to reduced overdose fatalities, lowered incidence of infections related to unsafe drug use (e.g., HIV and hepatitis C), and improved quality of life [9]. However, despite these well-documented benefits and recommendations from researchers, healthcare providers, and people who use drugs to scale-up SOS programs [8, 9], these programs are facing closures due to shifting provincial policies and impending federal funding expiration [14–16].

In August 2024, the provincial government proposed new restrictions affecting these services, first proposing zoning restrictions for CTS/SCS in Ontario in August [14], followed by Bill 223, *The Safer Streets*,* Stronger Communities Act*, in December 2024.^(15)^ The bill’s *Community Care and Recovery Act (CCRA)* component prohibits CTS/SCS within 200-metres of schools and childcare centres, citing concerns around public safety, community disruption, and visibility of drug use near these areas [15, 16]. While not explicitly banning SOS programs, the Act restricts municipalities from applying for federal funding or exemptions under Canada’s *Controlled Drugs and Substances Act (CDSA)* to support safer supply initiatives [15]. In addition, the CCRA requires sites to obtain provincial approval before participating in federally-funded CTS/SCSs, effectively forcing the closure of ten CTS/SCS across the province, six of which house SOS programs [14, 16]. Additionally, federal funding for remaining SOS programs expired March 31, 2025, with renewal requiring provincial approval under the CCRA [15]. Without renewed investment, these programs will also face closure, exacerbating the public health and social challenges already affecting people who use drugs .

Against this backdrop, the provincial government has committed to funding 29 Homelessness and Addiction Recovery Treatment (HART) Hubs, which focus on prevention, treatment and recovery and explicitly exclude harm reduction services, including CTS/SCS, SOS, and needle exchange programs [16]. By March 2025, nine of the ten CTS/SCSs received approval to convert to HART Hubs, effective April 1, 2025 [14]. The shift away from harm reduction raises serious concerns about service access for people who use drugs and contradicts evidence-based drug policy [8]. Indeed, this policy direction is at odds with the CDSS framework [5], that emphasizes a continuum of care, including harm reduction and comprehensive, evidence-based interventions for people who use drugs [5]. Despite overwhelming evidence that SOS programs reduce overdose deaths, infectious disease transmission, and other drug-related harms [5, 11, 12, 17], Ontario’s restrictions undermine core CDSS action areas.

Given the impending closures, this study aims to explore SOS clients’ perspectives on these closures and their impact on clients’ lives. It also seeks to understand how clients anticipate these closures will affect their health and well-being, and what coping strategies they may employ. By centering the voices of people who use drugs, this research provides critical insights into the consequences of policies that undermine evidence-based SOS programs, informing advocacy efforts and recommendations to mitigate harm within Ontario’s substance use strategy.

## Methods

This study employed a qualitative research design involving one-on-one semi-structured interviews (via telephone or Webex) with people who use drugs who were enrolled in a SOS program in Ontario at the time of the interview. Interview guides were developed in collaboration with our research team, including people with lived and living experiences. The guides explored participants’ experiences with SOS programs, their perceptions of the impact of program closures, and strategies they will undertake. This study received research ethics approval from the Centre for Addiction and Mental Health (CAMH) (REB #2024/173).

### Participant recruitment and eligibility

Convenience and snowball sampling were used to recruit participants. Study details and recruitment flyers with a toll-free number and study email address were shared through existing partnerships (i.e. Ontario Node of the Canadian Research Initiative in Substance Matters [OCRINT], provincial SOS sites) and circulated by staff at four participating SOS programs. Participants and partners were encouraged to refer eligible individuals from their networks.

Interested participants contacted the study team via email or the toll-free line and underwent a brief screening process to confirm eligibility. As part of the process, participants were asked to identify which SOS program they were enrolled in. Eligibility criteria included being a current SOS client enrolled for at least six months, being 18 years of age or older, and speaking English. Once eligibility was confirmed, participants received study details and proceeded with the consent process, during which verbal consent was obtained and documented on Research Electronic Data Capture (REDCap), a secure web application for managing and inputting study data.

### Data collection

Interviews occurred between January 27 and February 10, 2025. All interviews were conducted via telephone or virtually through videoconferencing (i.e., Webex) to allow for participant representation in real time from across the province. Interviews were audio-recorded for transcription purposes and began with a brief (~ 10-minute) socio-demographic survey to collect data on sex, age, ethnicity, and housing status. Additional information regarding drug use profiles, length of SOS engagement, medications provided as part of the SOS program, and wraparound services accessed through their program were also collected. All survey data were entered directly into REDCap. The survey was followed by a semi-structured interview (~ 30–60 min) and explored participants’ experiences with the program, any anticipated challenges and impacts on their lives considering prospective SOS program closures, and strategies they plan to adopt to either reduce drug-related risks or access other opioid medications in response to the program closures. Each participant was assigned a unique ID number to preserve confidentiality. Data were collected until saturation was achieved, where no new themes or patterns emerged from additional interviews [18].

Participants received $50 honorarium via Interac e-transfer upon completing the interview. For those without banking access, e-transfers were sent to a designated individual chosen by the participant on their behalf.

Reflexivity was embedded throughout the study design and data collection process. Team members engaged in regular discussions to reflect on how their positionalities, values, and professional experiences may influence interview dynamics, data interpretation, and coding. Interviews were conducted by a researcher with lived and living experience trained in trauma-informed and harm reduction principles to ensure participants felt respected and heard, and to foster trust and comfort in discussing sensitive topics. Having people with lived and living experiences involved in the research design and implementation also helped to bridge potential power imbalances between researchers and participants. This approach contributed to more open and authentic dialogue. These reflective practices informed the subsequent stages of data analysis.

### Data analysis

Participant survey response data were exported from REDCap into a Microsoft Excel file and analyzed descriptively. Basic frequency analysis was conducted to summarize responses to close-ended survey questions, including counts and percentages for each response category, allowing for a general understanding of patterns across the sample. Interview audio recordings were transcribed by a third-party transcription company. Transcripts were reviewed for accuracy and quality by a research team member and imported into NVivo (version 12) for qualitative data management and analysis.

A preliminary coding framework was developed by the research team which encompassed overarching themes on participants’ experience with SOS programs and the role it played in addressing opioid- and non-opioid related harms, drug- use patterns, and access to services. The framework also included themes related to participants’ strategies they were employing in response to the closures. The codebook framework was refined iteratively through regular team meetings, during which emerging themes were discussed, and discrepancies in interpretation were resolved. Thematic analysis was employed following Braun and Clarke’s approach [38], involving multiple rounds of coding to identify major themes and sub-themes within the data. To enhance data consistency and analytical rigour, three members of the research team independently coded a subset of transcripts, followed by discussions to resolve discrepancies and refine the codebook before it was applied to the full dataset.

To support the credibility of the findings, a form of member checking was incorporated, where key preliminary findings and interpretations were shared with two participants to confirm the resonance and relevance of emerging themes. Their feedback informed the final analysis and helped ensure that participant voices were accurately represented in the findings.

## Results

The study included 25 participants from six SOS programs across the province, all of which operated within larger organizations, such as CTS/SCSs (*n* = 2), community health centres (*n* = 3), or greater primary health care clinics (*n* = 1).

### Participant socio-demographics

Participants’ average age was 44.9 years (SD: 8.02). The gender distribution was evenly split between men (48%) and women (48%), with one participant identifying as non-binary (4%). The majority of participants (68%) identified as Indigenous (First Nations/Inuit/Metis), while 32% identified as White (European descent).

Nearly two-thirds (60%) were housed, just under one-third (28%) were unhoused, and 12% were couch-surfing or staying with friends/acquaintances.

*Participant Drug- Use Patterns*.

Over two-thirds (68%) of participants indicated that their drug of choice was non-prescribed street opioids, and the majority (72%) indicated that inhalation or smoking was their preferred route of administration.

### SOS program information

Participants had varying lengths of enrollment in their SOS program, with 52% enrolled for two years or less, 32% enrolled between two to four years, and 16% enrolled for more than four years.

The vast majority (96%) of participants received take-home doses. Most participants picked up their medication at a pharmacy (68%), typically once a day (56%).

Participants most commonly accessed harm reduction supplies (64%), housing/social support services (64%), and CTS/SCS (64%) through their SOS program. See Table 1 for a full list of services participants accessed.

The most commonly received medication was tablet hydromorphone (Dilaudid), with all participants (100%) indicating they obtained this medication through their program. Additionally, the majority of participants (80%) received slow-release oral morphine (SROM; Kadian). Table 1 provides a breakdown of medications distributed through the SOS program.

**Table 1:** .Services accessed and medications received by participants at SOS program

Services Accessed*	*N*	%
Harm Reduction Supplies/Education	14	64
Housing/Social Supports	14	64
CTS/SCS	14	64
Drug-Checking	12	54
Substance Use Counselling	9	41
Primary Care	5	23
Recreational/drop-in programming	3	14
Peer Support	1	4
SOS Medications Received**		
Tablet Hydromorphone (Dilaudid)	25	100
Slow-release Oral Morphine (SROM; Kadian)	20	80
SROM + Tablet Hydromorphone	18	72
Methadone	4	16
Other	3	12
* Hydromorph Contin (slow release)*	*1*	*4*
* Dexadrine*	*1*	*4*
* Methylphenidate* (*Foquest)*	*1*	*4*
Methadone + Tablet Hydromorphone	3	12
SROM + Tablet Hydromorphone + Other (Dexadrine/Foquest)	2	8
Methadone + Tablet Hydromorphone + SROM	1	4
Dilaudid + Other (Hydromorph Contin [slow release])	1	4

*Some participants accessed multiple programs

**Some participants received multiple SOS medications resulting in totals exceeding 25

### Qualitative findings

The interviews revealed invaluable insights into the experiences of clients enrolled in SOS programs and their concerns regarding the closures. The findings are categorized into two main themes: (1) The role and impact of SOS programs: current benefits and future consequences; and (2) coping with uncertainty and adapting to change in light of SOS programs closures.

### The role and impact of SOS programs: current benefits and future consequences

The impending closure of SOS programs has deeply concerned clients who rely on them for health, safety, and stability. Participants noted improvements in drug- use patterns, overdose risk, access to wraparound services, relationships with healthcare providers, and quality of life. They fear closures will force them back to the unregulated drug market, increasing risk of harm or death and cutting them off from essential services.

#### Drug-use patterns

Enrolling in SOS programs reduced participants’ reliance on the toxic street supply by ensuring predictable access to prescription medications. For instance, one participant described their complete cessation of fentanyl since enrolling in the program: *“As long as I’ve been on the program*,* I’ve never used fentanyl or any other pills.” (Man*,* a ge 58*,* s ix years in SOS program)*.

They emphasized the stability achieved by having a reliable supply that helped them avoid the unregulated market: “*When I go home*,* I don’t use. I don’t even think about it. When I go home*,* as long as I’ve got my meds*,* my prescription meds*,* I’m fine. That’s it.” (Woman*,* age 45*,* three years in SOS program*).

Some described moving away from injecting toward oral ingestion or inhalation due to the formulations available through the SOS programs, where the majority of participants were prescribed oral medications: *“I was using needles and stuff when I was doing fentanyl and mixed it a lot with crystal meth…I haven’t used needles for a long time now. So it’s [SOS programs] definitely changed the way I use drugs too.” (Woman*,* age 33*,* five years in SOS program)*.

Despite these gains, participants feared that the closure of SOS programs would leave them no choice but to return to unregulated opioids: *“I didn’t really have to do fentanyl*,* because the pills were getting me off [fentanyl]. But the pills are gone*,* I’m going to have to go back to the fentanyl.”* (*Man*,* age 40*,* two years in SOS program*).

Additionally, some anticipated that losing access to prescribed oral medications would force them back to the unregulated drug supply, and in turn, back to injecting, increasing their drug-related risks.

#### Overdose risk

Many saw SOS programs as life-saving, offering safety from the toxic street supply: *“I would be dead if it wasn’t for this program”* (*Man*,* age 56*,* two years in SOS program*), and *“I would say I’m still here*,* right? I’m not using now. Fentanyl probably would have killed me by now if I wasn’t on the SOS” (Woman*,* age 40*,* four years in SOS program)*. A key factor in reducing this risk, as described earlier, was the participants’ decreased reliance on the unregulated street supply, which led to a significant decline in overdose risk. Participants discussed after gaining access to prescribed pharmaceutical-grade medications, overdoses either significantly decreased or were eliminated altogether. This impact is underscored by the fact that over half (52%) of participants reported never experiencing an overdose since program enrollment.

They feared closures would lead to increased overdoses, often describing it as a “death sentence”. A few predicted resurgence in overdoses should they lose access to regulated prescription-grade opioids: “*Bodies will fall” (Woman*,* age 47*,* two years in SOS program)*, and *“I predict a wave of overdoses” (Man*,* age 45*,* two years in SOS program*). Another participant further elaborated on this fear by characterizing the closures as a systematic erasure of people who use drugs: *“This is basically… wiping off the drug addicts… killing off the drug addicts.” (Man*,* age 32*,* three years in SOS program)*.

Additionally, long-term SOS participants worried that their reduced tolerance would make a sudden return to the unregulated supply particularly dangerous. Many participants had been enrolled in the program for an extended period, with an average enrollment of almost three years, experiencing a gradual decline in their physiological tolerance to high-potency unregulated opioids such as fentanyl. As a result, they feared that if forced to return to the unregulated supply, their reduced tolerance would put them at a heightened risk of overdose, as described by the following participant:

*“When you see somebody who used fentanyl 6 months ago and they go to use fentanyl now*,* it’s completely different. So imagining people that have been on the program for 5 years going back to the street supply*,* they’re going to overdose right away.” (Non-Binary*,* age 44*,* four years in SOS program)*.

#### Access to wraparound services

Because many SOS programs were integrated within low-barrier sites (e.g. CTS/SCS), participants could also access safer consumption spaces, drug-checking, harm reduction supplies, and other social supports. However, participants expressed concern that the closure of SOS programs would severely disrupt their access to these services. For some, this disruption would stem from the simultaneous closing of both their SOS program and the CTS/SCS in which it was embedded. Others feared that even if the larger site remained open, they would be less likely to engage with its services once their SOS program was discontinued. Some participants noted that their utilization of these services was often a byproduct of their engagement with the SOS program, as they were already required to visit the site for prescriptions or care, and also felt a sense of community that was fostered as part of the SOS program.

A major benefit of SOS programming was consistent access to harm reduction supplies, with 64% of participants relying on these services, as described by the following participant:*“[The SOS program] really helped me with getting clean supplies. Before I’d have to buy needles and so I would be trying to use them a little bit longer than I should have*,* not replacing them every time. So it helped with that” (Man*,* age 42*,* two years in SOS program).* As a result, many participants feared returning to using in unsafe environments without on-site supervision and loss of access to harm reduction supplies, would significantly increase their risk of infections and other serious health complications, as described by the following participant:*“If they close the programs… people are just going to be dying of infections*,* injecting in multiple spots and it gets infected*,* using dirty needles*,* hepatitis*,* AIDS.” (Man*,* age 43*,* four years in SOS program)*.

In addition, over half of participants highlighted the value of on-site drug-checking services, which helped reduce overdose risk by identifying contaminants in unregulated drugs: “*You can go and get your shit tested at the safer supply place and know if [the drugs are] interlaced [sic] with anything or not. How many places can you go and you know if your stuff is pure or contaminated*,* right?” (Man*,* age 43*,* four years in SOS program)*.

They also valued the integration of CTS/SCS within SOS programs, which provided a safe and supportive environment for substance use, medical supervision, and access to wraparound services. Participants feared that losing these coordinated supports would force them to use in unsafe environments, increasing risks of overdose, violence, infections and other drug-related harms: “*[If the] SOS closes*,* lots of people are probably going to start using street drugs more again. And then not having a safe injection site – you know*,* a lot of those overdoses are going to happen on the streets in back alleys […]And also not having medical professionals nearby*,* close by*,* there could be more increase in like overdoses as well. And not having anybody – any like Narcan*,* or medical staff close by to watch these people while they’re having overdose is also going to not be good. (Man*,* age 39*,* two years in SOS program)* and *“I’m going to lose the ability to get my drugs tested or have somewhere safe to use. So*,* I’ll probably get robbed*,* raped*,* die hopefully.” (Woman*,* age 31*,* two years in SOS program)*.

Some also described how SOS programs facilitated connections to food, shelter, and emotional support through the program’s wraparound services:

*“I needed winter boots*,* they got them for me. They got me a winter coat. They’ve got me a couple pair of shoes. They give us food and juice every day. They’re always there to advocate for me*,* or talk to me*,* or like help me not feel like the world’s going to end.” (Man*,* age 44*,* one year in SOS Program)*.

Participants indicated they might lose these benefits without the SOS program, which will impact all aspects of their life:

*“If [*people who use drugs *] don’t have that ability to go [to the SOS]*,* they’re not going to go there just for housing because they’re going to be on the streets trying to figure out where they’re getting their fix. It’s affecting everything. It’s not just getting your Dilaudid*,* it’s your housing*,* your mental health. If you just want to see a counsellor or just talk to somebody*,* all of it.” (Woman*,* age 49*,* six years in SOS program)*.

#### Relationships with SOS program staff and healthcare systems

Participants valued trusting, non-judgmental relationships with SOS staff in contrast to negative experiences in traditional healthcare settings: “*You don’t have to lie about what you’re doing. Like with other medical professionals I feel like I’m going to get judged if I speak like the truth. But not here; I can just be honest about where I’m at and how I’m feeling. It’s the best care I can get.” (Woman*,* age 53*,* two years in SOS program)*.

Some referred to the programs as a “safer supply family,” indicating they could rely on staff for support and guidance, where SOS staff played a crucial role in providing emotional and social support:*“I didn’t feel like I had nothing left. You know*,* like I actually had something to live for*,* to look forward to. [SOS staff] helped me look forward to good things.” (Man*,* age 32*,* three years in SOS Program)*.

Participants particularly valued staff members with lived experience with drug use:

*“Half of the staff can relate to the drug addiction issues because they are sober now and they used to have a problem. And they’re super caring and like you feel their love*,* you know? They don’t have to say it*,* but the way they help take care of you*,* it’s amazing.” (Man*,* age 44*,* one year in SOS Program)*.

This was contrasted with negative encounters they faced in traditional healthcare settings, particularly hospitals, sometimes avoiding them even in life-threatening situations due to previous experiences of stigma and discrimination, as described:

“*I’ve seen people who are practically almost overdosing but they wouldn’t have gone to the hospital because they’re afraid of the hospital. So they’ll come into the clinic and somebody will sit there and monitor them and make sure that if they need oxygen*,* they’ll give them oxygen. They’ll take care of them*,* otherwise they would have died. (Man*,* age 45*,* two years in SOS program)*

As such, they feared closures would further destroy trust in healthcare programs or providers:

*“Those who survived overdose*,* I don’t think that they’re going to trust programming and services and supports. The healthcare system has been such a harmful system to people who use drugs that it took a lot for these doctors to build up our trust. People going in for appointments for the first month*,* month-and-a-half of safe supply*,* they’re like*,* “Is this for real? Can I actually trust my doctor? Is this a joke or is this a trick?” […] And so I think if this [SOS] gets torn away from people*,* how are they supposed to trust anything else that comes out?” (Non-Binary*,* age 44*,* four years in SOS program)*.

Furthermore, participants expressed deep concerns that the distrust stemming from the closure of SOS programs could lead to disengagement from healthcare systems. Many feared that without the client-centered care they received in SOS programs, they would encounter punitive, abstinence-based models that would further alienate them from seeking support:

*“I think that some people may go into other programs hoping that the relationship is the same and opening up only to be punished further and be further oppressed and stigmatized by abstinence-based mindsets and addiction programming. And I think that that will completely deteriorate their willingness to access healthcare and be truthful with healthcare providers about the realities of their lifestyles*,* their substance use*,* their relationships to drug-using communities.” (Non-Binary*,* age 44*,* four years in SOS program).*

#### Quality of life

Having a stable supply of opioids freed participants from constant drug-seeking and the criminalization and unpredictability which comes with buying and using illegal substances. This improved finances, relationships and mental health. Many described rebuilding family ties:

“*My marriage has gotten better. My relationship with my mom*,* which is my only real family that I have*,* it’s like a 180. She trusts me again. I never thought I’d see that day because I stole from her so much in my addictions. And so she actually respects me on a really good level. You can’t even put a price tag on that for me because she’s 80 and it’s like I don’t know how much time I have with her. But I was at least able to do that in her lifetime.” (Man*,* age 42*,* two years in SOS program).*

One participant described drug seeking as an immense burden prior to joining the program:

*“Having to purchase medications on the street was extremely stressful. And it was having another full-time job because I was having to track down people that had opiates that I could and wanted to take… So it was a full-time job trying to find somebody with Dilaudids or Kadians to purchase in order to maintain my dose. So I was literally working three jobs and one of those part-time jobs literally just paid for my medications. So I worked 20 hours a week just to be able to get by on my medications on top of working my full-time job.” (Non-Binary*,* age 44*,* four years in SOS program).*

The ongoing need to secure substances, often demanding significant time and effort, had previously caused participants persistent stress and anxiety. Many described how engagement in the SOS program provided a sense of psychological relief and stability, knowing they had reliable supply access:

“*It’s a constant searching for a high when I was getting high. You’re always looking for the next score. And now I can rest knowing that I don’t have to do that. I have my prescription and I may just go on living normally*,* how normal people do*,* just on my prescription.” (Woman*,* age 47*,* two years in SOS program).*

This reduction in drug-seeking was also directly related to a reduction in criminal involvement. For instance, one participant reflected on how their criminal engagement were driven by desperation:

*“Like I was boosting. I was car-hopping. I was doing all that. And when I got on the program*,* I wasn’t sick where I needed to go and do that. I was so sick*,* when you have nothing*,* it’s like you got no choice. Like you don’t care. And you go into the store*,* and you don’t care if you get caught. Your mind is just focused on that. How am I going to get better? How am I going to fix this? I wasn’t even caring if the beepers went off.” (Man*,* age 42*,* two years in SOS program).*

The possibility of returning to unregulated drugs provoked concerns about relapsing into criminal behavior: “*I don’t want to say*,* but I’d probably go back to stealing for [drugs] again.” (Man*,* age 39*,* two years in SOS program)*.

Others reported substantial emotional distress at the thought of losing SOS programs, sometimes including suicidal ideation: *“I’m so angry honestly because I haven’t thought about suicide in a long fucking time*,* but I’ve thought about suicide since – this is fucked. This is so fucked. How they can justify doing this” (Woman*,* age 31*,* two years in SOS program)*.

### Coping with uncertainty and adapting to change in light of SOS program closures

With closures looming, participants considered various strategies: switching to traditional OAT (e.g., methadone, buprenorphine/naloxone), seeking new prescribers, or tapering their SOS doses, either voluntarily or under staff-imposed reductions. Many described uncertainty and anxiety around these decisions.

#### Consideration of OAT: willing participation, limited choices, and misalignment with preferences

Some participants viewed OAT as a last resort, but most felt it did not meet their needs. They expressed concerns about the rigid structure of OAT programs, including being pressured to take specific medications like methadone or Sublocade, and the loss of autonomy over their care. Participants had previously engaged with OAT before joining SOS programs and described those experiences as largely negative. Only a small number of participants expressed willingness to return to OAT, seeing it as a proactive, though not preferred, strategy to manage the uncertainty surrounding the impending closure of their SOS program.

However, the majority of participants viewed receiving OAT on its own as a last resort rather than a preferred option. They felt as though they were being forced or pressured to engage in it as it was presented as the only viable option: *“They’re not really giving me a lot of options. That doctor wants to take away pretty much all meds… He wants me on Sublocade*,* and I don’t want Sublocade.” (Man*,* a ge 44*,* o ne year in SOS program);* and, *“I haven’t been able to decide [which treatment option] because there’s really no better choice” (Man*,* age 44*,* one year in SOS program).*

Concerns over the rigid structure, loss of autonomy, and punitive nature of OAT programs were widespread. Participants particularly highlighted strict guidelines such as frequent urine screening, inflexible dosing schedules, and the highly monitored, invasive settings of OAT programs as major barriers, viewing OAT more like punishment than treatment. One participant described the level of surveillance and control in these settings: *“There’s additional witness dosing they do twice a week and more frequent urine drug screenings. It’s much more invasive… there’s cameras in there*,* and it’s really kind of uncomfortable” (Man*,* age 42*,* three years in SOS program)*.

Several had prior negative experiences with methadone, with one participant particularly adamant about their refusal to re-engage with this treatment and considering relocating to avoid it:

*“I don’t want to go back on methadone. I’ve been on methadone so many fucking times*,* and it’s harder to get off than fucking fentanyl…. Honestly*,* I might just move to BC or fucking Portugal or something.” (Woman*,* age 31*,* two years in SOS program).*

Another participant described their experiences and fear of returning back to methadone:

“*I’ve been on and off methadone since*,* 2000-and – shit – ’08*,* ’09. And it’s like on methadone*,* off methadone*,* on methadone*,* off methadone*,* because it just doesn’t suit my needs. And the idea of going back into that rotation*,* methadone actually caused me more harm than it did good because it caused my depression to go up. It caused issues within my work. It caused issues with my family. It caused issues when I would go into other healthcare settings – because the moment I would say methadone*,* flags would raise.” (Non-Binary*,* age 44*,* four years in SOS program)*.

Participants overwhelmingly expressed that OAT programs were not designed to accommodate the realities of their lives due to limited flexibility in medication options and dosing schedules, coupled with stern program requirements which would create a cycle of failure rather than stability. One participant captured this concern stating, *“I think people will have to turn back to methadone*,* Suboxone*,* or Sublocade*,* because they’ll be in withdrawal. But the way that those programs run*,* being punitive*,* it’s not going to help them to be stable.” (Non-Binary*,* age 44*,* four years in SOS program).*

#### Self-versus staff-Imposed tapering

Facing closure, a few chose a gradual self-taper to maintain control, and minimize withdrawal symptoms, with one participant stating, *“In the last three*,* four months*,* I’ve already sort of tapered down a little bit… I just would feel more comfortable if I got to the position where I wasn’t needing as much as I was normally taking” (Woman*,* age 46*,* five years in SOS program).*

Others experienced abrupt dose cuts imposed by staff. For instance, one person reported having their dose reduced from 22 hydromorphone tablets to two tablets over the course of a few weeks.

As a result of these drastic staff-imposed dose reductions, participants experienced withdrawal, which pushed them toward high risk coping strategies, such as renewed street fentanyl use: *“Now that I’m tapering*,* I’m starting to go back to doing more fentanyl*,* that [the SOS program is] cutting me off the pills.” (Man*,* age 40*,* two years in SOS Program).* Some reported hospitalizations and a return to high-risk behaviors due to forced tapering: *“I started using again*,* I ended up in the hospital almost every other week because if they drop us two pills a week*,* and that’s two pills in the morning*,* two pills in the evening dose. So*,* I was feeling shitty*,* and so I would go out and just use anything like anything I could get my hands on. I wasn’t even being careful where I bought my stuff. I was just mad.” (Man*,* age 42*,* one year in SOS program).*

For some, losing access to their full prescription forced them into survival strategies to afford street drugs. One participant described resorting to sex work again as a direct result of tapering: *“Because of the tapering down*,* I’ve already started [escorting] again… to buy more street drugs” (Woman*,* age 31*,* two years in SOS program).*

#### Seeking alternative clinics or prescribers

A small number of participants had successfully secured and transitioned to alternative providers through family doctors or within other healthcare settings, for example, one participant who transferred their prescription to their family doctor, stated, *“I’m lucky enough to have my family doctor who is in [program name] and she’s taking over my safe supply. But not everybody has that opportunity” (Woman*,* age 66*,* three years in SOS program).*

One site integrated its SOS program into a primary care clinic, ensuring uninterrupted service: *“We’re just transitioning into the primary care part… anybody involved in the program isn’t transferring out of the building*,* just into a different part” (Woman*,* age 51*,* one year in SOS program)*.

While some participants were able to secure alternative providers, the majority were left with uncertainty regarding their future access to SOS prescriptions.

#### Navigating uncertainty: coping with unclear plans

Many felt intense anxiety and had no definitive plan for when their programs ended. These participants expressed feelings of helplessness and frustration about their future medication access, as one participant shared:

*“I don’t even like to think about it because it’s just*,* I don’t know what I’m going to do. It’s the unknown and it’s so bad it’s giving me anxiety. I get anxiety attacks again all the time. Can’t sleep at night. I mean*,* it’s been horrible not knowing what’s happening or if I’m going to be able to go on with my prescription*,* living life. I don’t want to go back to drugs but I have a feeling I will if it comes down to me not getting a prescription. I don’t know*,* I can’t see myself living completely sober.” (Woman*,* age 47*,* two years in SOS program).*

Some described the limited and few viable options as destabilizing:

*“I just had a doctor’s appointment downstairs about what to do*,* and I’m running out of time to check. And like they’re not really giving me a lot of options. Like that doctor wants to take away pretty much all meds*,* and like it’s just going to end up putting me back where I was.” (Man*,* age 44*,* one year in SOS program).*

Several questioned their ability to survive if forced back to old patterns: *“I can’t go back to what I was before*,* I won’t make it”* (*Woman*,* age 46*,* five years in SOS program).*

## Discussion

The findings from this study add to the growing body of evidence highlighting the vital role SOS programs play in the lives of people who use drugs. These programs serve as a critical safeguard against the toxic, unregulated drug supply, while helping reduce reliance on street drug use, and fostering improvements in health, well-being, and engagement with essential health and social services. Beyond access to safer supply, these programs offered community connections and wraparound supports. As such, the forced closures of these programs across the province have sparked deep fear and uncertainty among participants, many of whom worried that these closures would reverse the hard-won progress they had made toward safety, stability, and recovery.

### A foreseeable public health catastrophe

Participants emphasized that SOS programs fundamentally transformed their drug- use patterns, leading to a reduction in fentanyl use, a shift away from injection drug use, and increased overall stability. These findings align with existing research showing that access to a regulated supply reduces engagement with the toxic drug market and decreases fentanyl use, the primary driver of overdoses in Canada [17]. Similar outcomes have been reported in Ontario SOS program evaluations [10, 11], with 86% of Peterborough clients and 82% of Kitchener-Waterloo clients reducing or stopping fentanyl use after 12 and 6 months of enrollment, respectively [12, 19].

With the dismantling of SOS programs, participants expressed fears of a rapid loss of these benefits, anticipating that they would be pushed back into the unregulated drug market, heightening their risk of overdose and death. As participants began tapering off their SOS medications, whether through gradual, self-initiated tapering or rapid staff- imposed dose reductions, these risks began to materialize. Many reported returning to the unregulated market and engaging in survival strategies, such as sex work, while others noted a rise in hospitalizations following the reduction or cessation of their medication. Participants expressed concern that their prolonged access to prescribed alternatives had significantly lowered their opioid tolerance, making renewed exposure to the unregulated supply particularly dangerous. Previous research supports these concerns, showing that individuals who experience disruptions in SOS programs are at increased risk of fatal overdose due to lowered tolerance and exposure to an increasingly toxic drug supply [20]. For instance, a study on SOS program discontinuation in Ottawa found that the abrupt loss of safer supply medications and subsequent return to unregulated and highly toxic street market triggered a rise in overdoses and other associated harms [21].

Beyond access to safer supply, SOS programs provided participants with an entry point to essential services, including housing supports, CTS/SCS, drug checking, primary care and mental health resources. Research has consistently shown that low-barrier harm reduction programs enhance engagement with healthcare and social services, by offering accessible, non-judgmental, and supportive environments [8, 17]. These programs often operate as integrated one-stop service hubs, reducing barriers to care, and facilitating connections to essential services [22]. Participants highlighted that SOS programs increased their engagement with health and social services through the development of trusting relationships with service providers. Many echoed concerns with the closure of SOS programs, describing past experiences of stigma and discrimination in conventional healthcare settings that deterred them from seeking care. SOS programs, by contrast, have provided people who use drugs with a safe, supportive, and non-judgmental environment, enabling access services without fear of stigma or discrimination [23].

Participants feared that without SOS programs, they would become disconnected from these vital resources, either forcefully due to the closures, or because they would feel less likely to access these programs once their SOS program is discontinued. Similar disruptions have led to a number of health and social implications, as seen in studies examining the consequences of harm reduction service fragmentation [24, 25]. For example, the closure of a fixed needle exchange site and the subsequent opening of a mobile needle exchange van in Vancouver led to a 40% reduction in needle distribution and recovery rates, meaning more needles were left unaccounted for, potentially increasing unsafe practices such as needle sharing or reuse [25]. Similarly, the closure of the ARCHES SCS in Lethbridge, Alberta, the busiest CTS/SCS in North America at the time, resulted in a surge of public drug use, increased emergency service calls, and an overall deterioration of health and social supports for people who use drugs [26]. These findings echo concerns expressed by participants, that disrupting harm reduction services will lead to decreased engagement with life-saving services.

### Economic and social costs of harm reduction programs

Participants anticipated a surge of overdoses and subsequent hospitalizations following SOS program closures, placing even greater strain on an already overwhelmed healthcare system both financially and physically [8]. In Ontario, opioid-related emergency department visits and hospitalizations have risen sharply, with 12,527 opioid-related emergency department (ED) visits and 2,064 hospitalizations in 2020 alone, costing an estimated $11.39 million, and $33.18 million, respectively [1, 27]. These figures underscore the immense financial and public health burden of the opioid crisis and signal the added strain that dismantling SOS programs could play on an already overwhelmed healthcare system.

These anticipated outcomes parallel the devastating consequences seen during the COVID-19 pandemic, when disruptions to harm reduction services similarly increased financial and physical burdens on the healthcare system [28, 29]. For example, widespread reductions in access to mental health, addiction, and harm reduction services led to increased opioid-related deaths and hospitalizations, increasing the strain on the healthcare system [29]. The direct financial cost of opioid-related harms, including hospitalizations, paramedic calls, ED visits, and deaths, rose from $162 million in 2018 (pre-pandemic) to $181.77 million in 2020 (pandemic period), underscoring the substantial economic and public health impact of failing to maintain accessible harm reduction services [27]. Given that harm reduction initiatives, such as CTS/SCS, have been shown to generate substantial healthcare savings, with successful overdose reversal estimated to save the healthcare system approximately $35 million over five years, these closures are not only a public health risk but also a costly policy decision [30].

### A shift away from harm reduction and lack of evidence

Amid a broader shift away from harm reduction, the Premier of Ontario has promoted harmful rhetoric, most notably referring to the federal government as a “drug dealer” for supporting evidence-based initiatives like safer supply [31]. Such statements reflect a fundamental misunderstanding of the goals and proven outcomes of these programs. Rather than addressing the root causes of drug-related harms, this narrative has been used to justify a return to punitive approaches that have consistently failed to reduce drug-related harms [32].

Public safety concerns and visible public drug use have frequently been cited as justification for scaling back SOS programs. However, evidence from Toronto, Ontario, has shown that the integration of CTS/SCS has been associated with declines in assaults, robberies, and homicides in surrounding neighbourhoods [33–35]. Additionally, access to these services significantly reduces public drug use, illustrated by a 50% decrease in high frequency public drug use among individuals experiencing homelessness or unstable housing in Toronto [33, 34]. As such, these closures may in fact exacerbate these very issues, leading to increased crime and public drug use, outcomes directly contradicting this rationale [33, 40]. With the closures looming, many participants expressed fears that they would be forced to return to engaging in criminal activity as a means for obtaining unregulated drugs.

Additionally, concerns about medication diversion have also been cited as justification to dismantle such programs, however, further research is needed to better understand the extent and context of diversion within safer supply models, especially as some diversion may occur as a form of harm reduction [36, 37]. For instance, a study examining diversion within SOS programs found that some participants shared portions of their prescribed medications with peers to help manage withdrawal symptoms or reduce their risk of overdose [36]. Despite this, SOS program closures are largely being framed as necessary to curb diversion, not appropriately weighing the risks and benefits of these closures.

Ontario is not alone in scaling back safer supply programs without robust empirical evidence to justify these decisions, British Columbia (BC), once a forerunner in harm reduction initiatives, has also begun rolling back harm reduction initiative, such as safer supply programs [38, 39]. As of February 2025, individuals enrolled in SOS programs in BC must now consume their prescribed medication under direct supervision by a pharmacist or health care provider [40]. Notably, in Alberta, a 2019 government-commissioned review assessed the socioeconomic impacts of 7 SCSs [39]. While presented as an objective evaluation, the report was fundamentally methodologically flawed, with a high risk of biases that critically undermined its assessment of the scientific evidence. Nevertheless, its findings have been used to justify decisions that jeopardize the health and well-being ofpeople who use drugs both in Canada and internationally.

Overall, some of the justifications for dismantling SOS programs do not align with research highlighting that harm reduction programs have led to a reduction in criminal involvement and public drug use, which was echoed by participants in this study. As a result, it is imperative to conduct long- term evaluations of SOS programs grounded in a comprehensive review of all the evidence to adequately inform policy changes.

#### A call for policy reform: integration of treatment and harm reduction

A total of 27 Hart Hubs have been approved in Ontario and are scheduled to open as of April 1st 2025, nine of which will replace existing CTS/SCS which are set to close on March 31st, 2025 [41]. However, as the Hart Hubs are not yet operational, it remains unclear how both sites and clients will transition to this new model of care [8], especially since Hart Hubs will exclude core harm reduction services, such as SOS programs, CTS/SCS, and needle exchange [8, 16]. Although Hart Hubs aim to enhance access to treatment and are presented as an alternative to CTS/SCS, this model falls short of what CTS/SCS offer. Hart Hubs lack critical harm reduction services (safer supply, drug checking, supervised consumption) and fail to recognize that CTS/SCS sites already provide access and referrals to a wide range of treatment services, and have been proven effective in reducing overdose deaths, fostering healthcare engagement, and serving as a critical entry point to care for people who use drugs [7–12, 19, 27, 32, 42, 43]. For instance, evidence from Wood et al. (2007) showed that individuals who used a supervised injection facility were 1.7 times more likely to enroll in a detoxification program compared to those who did not, with many reporting that their engagement with the SCS directly facilitated access to addiction treatment services [44]. By removing harm reduction services, such as safer supply, the Hart Hubs ignore one of the most effective tools in preventing overdose and stabilizing lives, and risks severing these critical points of connection. Further, the closure of harm reduction programs reinforces the distrust people who use drugs have toward healthcare systems, and undermining years or progress made in engaging people who use drugs in care [45].

Recognizing these concerns, many organizations across Ontario, including the Canadian Mental Health Association, have urged the government to implement the Hart Hub model alongside, rather than in place of, existing harm reduction services [46]. While treatment options, such as OAT, remain valuable for many people who use drugs, many participants in this study shared that they had previously attempted OAT but did not find it effective or suitable for their needs. They highlighted concerns with treatment services, discussing how the punitive, inflexible nature of these programs are not accommodating to their lives. This underscores the importance for developing treatment alongside harm reduction programs, as replacing harm reduction programs with treatment options will not promote individuals to access treatment. Instead, participants discussed that with the impending closures, they feared a return to the unregulated market. Notably, the legislation introduced by the provincial government has been challenged in court by an organization which runs one of the CTS/SCS slated for closure, *The Neighbourhood Group*, as a potential violation of the Constitution and the Charter of Rights and Freedoms [47]. While the sites slated for closure have been permitted to remain open temporarily for an additional 30 days after the judge has made a decision regarding the case, pending further evidence and clarification from the government regarding the closures of CTS/SCS and the implementation of Hart Hubs, nine of these sites have already shut down.

Moving forward, a comprehensive and integrated approach is needed, one that scales up SOS programs and balances both harm reduction and treatment models, especially as many participants highlighted that treatment services were not accommodating to their lifestyle, which deterred many from accessing this service, ultimately forcing them back to the unregulated market.

## Limitations

This study has several limitations. First, while the insights gathered provide valuable perspectives on the impact of SOS program closures, the sample size was limited to 25 participants. As a result, findings may not be generalizable to all people who use drugs in Ontario, nor can they fully capture the broader community-level impacts of SOS program closures. Rather, the study highlights individual experiences and perceived consequences among participants who were directly engaged with these services. Second, the study relied on self-reported data, which is subject to potential bias, including recall bias and social desirability bias. Participants may have framed their experiences in ways that align with perceived expectations or may have under or over-reported certain behaviors. Finally, as the study was conducted shortly after the announcement of SOS program closures, it captures only a snapshot in time. The longer-term effects of these closures on drug- use patterns, health outcomes, or engagement with alternative services such as OAT or HART Hubs remain unknown and warrant future research.

## Conclusion

Study findings highlight an urgent need to maintain and expand SOS programs as part of Ontario’s overdose response. Participants consistently discussed how SOS programs reduced their overdose risk, facilitated access to health and social services, fostering trusting relationships with staff and healthcare providers, and significantly improved their quality of life. With the impending closures, participants expressed deep concerns about being forced to return to the unregulated drug market, placing them at greater risk of overdose, disconnection from vital services, and a lack of viable alternatives to support their health. Additionally, forced tapering from SOS medications was described as physically and emotionally destabilizing, driving many back to the unregulated market. Rather than reducing drug use or improving public safety, participants feared closures would drive them toward criminalized behaviors to afford drugs and increase public drug use due to a lack of safe, private consumption spaces. Dismantling SOS programs under the guise of reducing public drug use fails to address the underlying demands for opioids or offer safer alternatives. Instead, it penalizes individuals who have actively stabilized their opioid use through evidence-based programming and forces them back into high-risk, unregulated environments. SOS programs represent more than access to safer medications, they provide stability, dignity, and hope. Participants urged governments to expand, not eliminate, SOS programs alongside new initiatives like Hart Hubs to create a comprehensive, integrated system of care. Without this, the current policy direction risks a devastating setback in the fight against the overdose crisis, one that will be measured in preventable harms and lives lost. Long- term evaluations and assessments of SOS programs are needed, and must be based on empirical and methodologically sound evidence.

## Data Availability

No datasets were generated or analysed during the current study.

## Abbreviations

BC: British Columbia
CAMH: Centre for Addiction and Mental Health
CCRA: Community Care and Recovery Act
CDSA: Controlled Drugs and Substances Act
CDSS: Canadian Drugs and Substances Strategy
CTS: Consumption Treatment Services
ED: Emergency Department
Hart Hubs: Homelessness and Addiction Recovery Treatment Hubs
OAT: Opioid Agonist Treatment
OCRINT: Ontario Node of the Canadian Research Initiative in Substance Matters
REDCap: Research Electronic Data Capture
SCS: Supervised Consumption Services
SOS: Safer Opioid Supply
SROM: Slow-Release Oral Morphine
SSP: Safer Supply Program
SUAP: Substance Use and Addictions Program
